# Oral Vaccination of Largemouth Bass (*Micropterus salmoides*) against Largemouth Bass Ranavirus (LMBV) Using Yeast Surface Display Technology

**DOI:** 10.3390/ani13071183

**Published:** 2023-03-28

**Authors:** Mengjie Zhang, Xiaoyu Chen, Mingyang Xue, Nan Jiang, Yiqun Li, Yuding Fan, Peng Zhang, Naicheng Liu, Zidong Xiao, Qinghua Zhang, Yong Zhou

**Affiliations:** 1 National Demonstration Center for Aquatic Animals, Shanghai Ocean University, Shanghai 201306, China; 2 Yangtze River Fisheries Research Institute, Chinese Academy of Fishery Sciences, Wuhan 430223, China; 3 Key Laboratory of Exploration and Utilization of Aquatic Genetic Resources, Ministry of Education, Shanghai Ocean University, Shanghai 201306, China; 4 Anhui International Travel Health Care Center, Hefei Customs, Hefei 230061, China

**Keywords:** largemouth bass ranavirus, oral vaccine, *Saccharomyces cerevisiae*, yeast display technology, mucosal immunization

## Abstract

**Simple Summary:**

LMBV is an important pathogen in the breeding process of largemouth bass, but there is no effective control method for it. This study developed oral vaccines for LMBV. The vaccines can significantly increase the activity of immune-related enzymes, upregulate the expression of immune genes, and stimulate the production of neutralizing antibodies in serum in largemouth bass. In addition, the vaccines can reduce the mortality of LMBV infection. These results indicate that the vaccines are expected to be candidate vaccines for controlling LMBV infection.

**Abstract:**

Largemouth bass ranavirus (LMBV) infects largemouth bass, leading to significant mortality and economic losses. There are no safe and effective drugs against this disease. Oral vaccines that directly target the intestinal mucosal immune system play an important role in resisting pathogens. Herein, the B subunit of *Escherichia coli* heat-labile enterotoxin (LTB, a mucosal immune adjuvant) and the LMBV main capsid protein (MCP) were expressed using *Saccharomyces cerevisiae* surface display technology. The yeast-prepared oral vaccines were named EBY100-OMCP and EBY100-LTB-OMCP. The candidate vaccines could resist the acidic intestinal environment. After 7 days of continuous oral immunization, indicators of innate and adaptive immunity were measured on days 1, 7, 14, 21, 28, 35, and 42. High activities of immune enzymes (T-SOD, AKP, ACP, and LZM) in serum and intestinal mucus were detected. *IgM* in the head kidney was significantly upregulated (EBY100-OMCP group: 3.8-fold; BY100-LTB-OMCP group: 4.3-fold). *IgT* was upregulated in the intestines (EBY100-OMCP group: 5.6-fold; EBY100-LTB-OMCP group: 6.7-fold). Serum neutralizing antibody titers of the two groups reached 1:85. Oral vaccination protected against LMBV infection. The relative percent survival was 52.1% (EBY100-OMCP) and 66.7% (EBY100-LTB-OMCP). Thus, EBY100-OMCP and EBY100-LTB-OMCP are promising and effective candidate vaccines against LMBV infection.

## 1. Introduction

Largemouth bass (*Micropterus salmoides*), also known as California bass, have the characteristics of fast growth, resistance to low temperatures, delicious meat, and easy fishing [1]. Largemouth bass was introduced into China in the 1980s and has since become an important freshwater aquaculture species [2]. However, with the expansion of the breeding scale and the increased stocking density, problems related to bacterial, virus, and parasite infections are becoming more prominent [3,4,5]. Among them, the disease caused by Largemouth bass ranavirus (LMBV) infection is the most serious.

LMBV, a strain of the *Santee Cooper ranavirus*, belongs to the genus *Ranavirus* of the *Iridoviridae* family, comprising a naked or enveloped icosahedral virus with double-stranded DNA [6,7]. LMBV was first reported to cause massive mortality of largemouth bass in the 1990s [8]. At present, LMBV has spread to many places (South Carolina, Texas, New York, Arkansas, Guangdong, etc.) [3,9,10,11,12]. The disease caused by LMBV infection is difficult to diagnose by signs and symptoms. Infected largemouth bass may lose their balance and float on the water surface [8]. Some Asian strains of LMBV can cause extensive ulceration of the fish body surface, muscle necrosis, and spleen and kidney swelling [3,13]. Healthy fish generally carry LMBV, which can lead to extensive virus spread [14]. Among the largemouth bass without signs of disease from 37 different locations in New York State, samples from 13 locations carried LMBV [9]. Currently, there is no effective prevention or treatment for LMBV infection.

Vaccination is considered an important means of controlling aquatic animal diseases [15]. The LMBV main capsid protein (MCP) has virus-specific surface antigens. The MCP is often used as a target protein for vaccine research in the laboratory [16,17]. Using the *MCP* gene as the target sequence, a DNA vaccine for LMBV was constructed and inoculated by pectoral fin base injection, which could induce a significant immune response in largemouth bass [18]. The vaccine prepared by expressing the LMBV MCP protein in *Pichia pastoris* resulted in a relative percent survival (RPS) of 41.6% against LMBV infection [19]. These results indicated that the expression of LMBV MCP could protect largemouth bass against LMBV infection.

*Saccharomyces cerevisiae* is the most commonly used yeast for recombinant protein production [20]. Recombinant drugs derived from eukaryotic microorganisms approved by the American Food and Drug Administration (FDA) and the European Medicines Agency (EMEA) are almost all produced by *S. cerevisiae* [21], such as the Hepatitis B vaccine (HBV) commercial vaccine Recombivax HB (Merck and Co., Inc., Whitehouse Station, NJ, USA) and the human papillomavirus (HPV) commercial vaccine Gardasil (Merck and Co., Inc.) [22,23,24]. Immune adjuvants can markedly increase vaccine efficacy [25]. The B subunit of *Escherichia coli* heat-labile enterotoxin (LTB) is considered a potent oral adjuvant that boosts immune responses when co-administered with antigens [26]. The main mechanism of its immune adjuvant activity is binding to ganglioside GM1 of mucosal epithelial cells [27]. In this study, the *MCP* gene and an *ltb*-*MCP* fusion gene were expressed separately in *S. cerevisiae* to prepare oral vaccines. To evaluate the immune effect of the oral vaccines, we measured the activity of immune-related enzymes and detected the expression levels of immune-related genes. The serum neutralizing antibody titers and the RPS after LMBV challenge were evaluated. The aim was to provide a safe and effective solution for the prevention of LMBV infection.

## 2. Materials and Methods

### 2.1. Virus, Cell Lines, and Fish

LMBV and epithelioma papilloma cyprinid (EPC) cells were obtained from the Yangtze River Fisheries Research Institute, Chinese Academy of Fishery Sciences (Wuhan, China) [28]. EPC cells were maintained at 25 °C in M199 medium (Hyclone, Logan, UT, USA) supplemented with 10% fetal bovine serum (FBS). Healthy largemouth bass (25 ± 5 g) without LMBV were purchased from a certified largemouth bass farm in Wuhan City, Hubei Province, China. Fish were temporarily reared in our laboratory’s recirculating aquaculture system. During the breeding period, fish were fed with commercial feed (≥48% protein, ≥6% fat, ≤12% water, ≤12% ash, Tongwei, Chengdu, China) at 9 a.m. and 6 p.m., and the feeding amount was 2% of the body weight of the fish. The water temperature was 26–28 °C, and the water was changed every other day. All animal experiments were approved by the Animal Experimental Ethical Inspection of Laboratory Animal Centre, Yangtze River Fisheries Research Institute, Chinese Academy of Fishery Sciences (ID Number: YFI 2022-zhouyong-07-3).

### 2.2. Strains and Plasmids

*Escherichia coli* DH5α (Takara, Taejin, Japan) cultured in Luria-Bertani (LB) medium (Solarbio, Beijing, China) at 37 °C was used for vector construction. *Saccharomyces cerevisiae* EBY100 (Invitrogen, Carlsbad, CA, USA) was grown in yeast peptone dextrose adenine (YPDA) medium at 30 °C (Biosharp, Beijing, China). Recombinant yeasts were screened on minimal dextrose plates (MDP) containing 2% glucose (0.67% amino-free yeast nitrogen source medium (YNB), 0.01% leucine) (Biosharp) at 30 °C. Positive transformants were cultured at 30 °C on YNB-CAA medium (0.67% YNB, 0.5% acid-hydrolyzed casein) containing 2% glucose (Biosharp). Recombinant protein expression was induced on the YNB-CAA medium containing 2% galactose at 20 °C in the dark. The pYD1 vector (Invitrogen) was used to express proteins in yeast cells. The pCR2.1-LTB expression vector to amplify the *ltb* gene was stored in our laboratory [29].

### 2.3. Codon Optimization and Gene Synthesis

The *MCP* gene sequence of LMBV (GenBank: MK836319.1) was codon-optimized for *S. cerevisiae* expression using the online software Java Codon Adaptation Tool (JCat) server (https://www.jcat.de/, accessed on 21 February 2022) [30]. The optimized parameters mainly included the codon adaptation index (CAI) and GC content. The optimized *MCP* gene was named *OMCP*. The *OMCP* sequence was synthesized by Huayu Gene Company (Wuhan, China).

### 2.4. Construction of the Recombinant Plasmids

*OMCP* was amplified using *OMCP* primers with *Eco*R I (forward primer) and *Not* I (reverse primer) restriction enzyme sites (NEB, Beijing, China). The amplified product was inserted into the pYD1 vector using recombinase (Vazyme, Nanjing, China) to obtain pYD1-*OMCP*. The *ltb* primers containing *Acc*65 I (forward primer) and *Bam*H I (reverse primer) restriction enzyme sites were designed according to the *ltb* sequence (GenBank: M17874.1). The *ltb* sequence was amplified from the pCR2.1-LTB plasmid and inserted into pYD1-*OMCP* to obtain pYD1-*LTB*-*OMCP*. The correct recombinant plasmids were identified using PCR. The primers used are shown in Table 1.

### 2.5. Screening and Induction of Expression in Vaccine Strains

Recombinant plasmids pYD1-*OMCP* and pYD1-*LTB*-*OMCP* were transformed into *S. cerevisiae* competent cells using electroporation [33]. Competent cells were plated on MDP and cultured at 30 °C (CIMO, Shanghai, China) for 2–3 d until colonies appeared. A single colony was picked, placed in 50 μL of distilled water, and then boiled for 10 min. The boiled colony suspension was used as a template for PCR analysis. After identification via PCR, the positive colony was inoculated into 10 mL 2% glucose YNB-CAA medium and sharking cultured at 30 °C and 220 rpm (New Brunswick Scientific, Edison, NJ, USA). When the optical density at 600 nm (OD_600nm_) of the yeast reached 2–5, the yeast cells were collected by centrifugation (1000× *g*, 5 min) (Eppendorf, Hamburg, Germany). The cells were then suspended in 2% galactose YNB-CAA medium, and the OD_600nm_ was adjusted to 0.5–1. The yeast was cultured at 20 °C in dark (220 rpm, 48 h).

### 2.6. Immunofluorescence Analysis

The expression of EBY100-OMCP and EBY100-LTB-OMCP was induced by galactose in the dark. A total of 1 mL of yeast (OD_600nm_ = 1) was harvested by centrifugation (4 °C, 3000× *g*, 5 min). The yeast cells were washed three times with sterile phosphate-buffered saline (PBS; Hyclone, Logan, UT, USA). After centrifugation (4 °C, 3000× *g*, 5 min), we added 500 μL murine 6 × His-tag antibody (1:1000) (ab18184, Abcam, Cambridge, UK) to the cells and incubated them at room temperature for 2 h. The cells were centrifuged again and washed three times with sterile PBS plus 0.05% Tween 20 (PBST). Then, 500 μL of goat anti-mouse IgG-H&L (Alexa Fluor 594) secondary antibody (1:500) (A-11005, Invitrogen) was added, and the cells were incubated in the dark for 1 h at room temperature. Yeast cells were washed three times with sterile PBST and resuspended in PBS. A small amount of the yeast suspension was coated on a sterile glass slide. Slides were subjected to confocal microscopy (Olympus, Tokyo, Japan) for imaging.

### 2.7. Detection of Recombinant Yeast in the Intestines

The recombinant yeast in the second intestinal segment (midgut) of the largemouth bass was detected using immunohistochemistry. Fish received 100 μL of recombinant yeast EBY100-OMCP or EBY100-LTB-OMCP (OD_600nm_ = 1) via gastric gavage (*n* = 5). After 24 h, the midgut of the fish was excised under anesthesia with MS222 (100 mg/L, Sigma, St. Louis, MO, USA) and fixed in 4% paraformaldehyde universal tissue fixative (Servicebio, Wuhan, China). Frozen sections were prepared according to a previous publication [34]. Intestinal frozen sections were incubated overnight at 4 °C with primary antibodies (1:500, anti-murine 6 × His-tag) (ab18184, Abcam) and then incubated in the dark at room temperature for 2 h with secondary antibodies (1:500, Alexa Fluor 594) (A-11005, Invitrogen). 4′,6-diamidino-2-phenylindole (DAPI) (Sigma) was then used for nuclear staining. Finally, the yeast cells were observed under a fluorescence microscope (Olympus).

### 2.8. Oral Immunization and Sample Collections

The recombinant yeasts EBY100-OMCP and EBY100-LTB-OMCP were induced to express the recombinant proteins in large quantities. Yeast cells were harvested using centrifugation (4 °C, 3000× *g*, 5 min). We then added 5% (*w*/*v*) skimmed milk powder and 50% (*v*/*v*) water to the yeast cells; 5% (*w*/*v*) starch was used as a binder. Fully mixed yeast and feed (1 × 10^7^ colony forming units (CFU)/g) were dried overnight in a freeze dryer (SCIENTE, Ningbo, China) to obtain the oral feeding vaccines. Healthy largemouth bass were randomly divided into four groups, namely the control (ordinary feed), EBY100-pYD1 (empty vector yeast feed), EBY100-OMCP (MCP vaccine), and EBY100-LTB-OMCP (LTB-MCP vaccine). For each group of 180 fish, the feeding amount was 2% of the body weight of the fish, twice a day, for continuous oral immunization for 7 days. The sampling time points were 1, 7, 14, 21, 28, 35, and 42 days post-immunization (dpi). At each sampling time point, largemouth bass (*n* = 5) were anesthetized using MS222. Blood samples were taken from the tail vein, placed overnight at 4 °C, and then centrifuged at 4 °C, 5000× *g*, for 10 min. The obtained supernatant (serum) was stored at −80 °C for subsequent neutralization antibody titer determination and enzyme activity assessment. The intestinal mucosa of the midgut was excised and preserved at −80 °C for enzyme activity determination and immune-related gene analysis. In addition, different intestinal parts (foregut, midgut, and hindgut) were sampled at 21 dpi and placed in a 4% paraformaldehyde universal tissue fixative for histological observation. The vaccination and sampling schedule are shown in Figure S1.

### 2.9. Vaccine Safety Evaluation

The foregut, midgut, and hindgut tissue sections of largemouth bass at 21 dpi were observed using an ordinary optical microscope at 20× magnification (Olympus). The tissues were fixed in 4% paraformaldehyde universal tissue fixative, followed by dehydration, transparent, wax penetration, paraffin embedding, and sectioning at 5 μm. After hematoxylin-eosin (H&E) staining and neutral resin sealing, the sections were observed and analyzed under the microscope. During immunization, the feeding and activity of the fish in each group were observed daily.

### 2.10. Determination of Immune-Related Enzyme Activity

The activities of superoxide dismutase (SOD), alkaline phosphatase (AKP), acid phosphatase (ACP), and lysozyme (LZM) in the serum and intestinal mucosa of the largemouth bass were determined according to the instructions of the enzyme activity kits (Jiancheng, Nanjing, China). The intestinal mucosa was collected according to a previously described method [35]. The intestinal mucosa was weighed and diluted with sterile PBS to a 1% tissue homogenate concentration and centrifuged at 4 °C, 5000× *g*, for 20 min. The obtained supernatant was used for subsequent enzyme activity determination. Based on the Bradford method, bovine serum albumin (BSA) was used as the standard to determine the total intestinal protein content [36]. Each group of samples was repeated three times.

### 2.11. Expression of Immune-Related Genes

Total RNA was extracted from the head kidney and intestine of largemouth bass using the Trizol reagent (Invitrogen). cDNA was synthesized from the RNA using a reverse transcription kit (TransGen Biotech, Beijing, China) and stored at −20 °C. The qPCR reactions for four immune genes (*TNF-α* (encoding tumor necrosis factor alpha), *IL-1β* (encoding interleukin 1 beta), *IgM* (encoding immunoglobulin M), and *IgT* (encoding immunoglobulin T)) in all samples were completed using a qPCR instrument (Corbett, Sydney, Australia). The reaction program was 95 °C for 10 min, followed by 40 cycles of 95 °C for 30 s and 60 °C for 30 s. The reaction system comprised 2 μL of diluted cDNA sample, 10 μL of Hieff qPCR SYBR Green Master Mix (Yeasen, Shanghai, China), 0.8 μL of forward primer and reverse primer (10 M), and 6.4 μL sterile H_2_O. Gene expression analysis was performed using the 2^−ΔΔCT^ method, with the *β-actin* gene as the internal control gene for cDNA normalization [37]. The primers used are shown in Table 1. All the experiments were repeated three times.

### 2.12. Serum Neutralization Antibody Assay

Serum neutralizing antibody titers were determined using LMBV and EPC cells [38]. The serum samples were defrosted at 4 °C and then heated at 56 °C for 30 min. The serum was then serially diluted (1:10, 1:20, 1:40, 1:80, 1:160, 1:320, 1:640, and 1:1280) with M199 medium (without FBS). A total of 50 μL of serum at different dilutions and 50 μL of 10^3^ TCID_50_/mL LMBV virus were mixed in 96-well plates. Six duplicate wells were set for each serum dilution concentration, and positive and negative control wells were also set. After gentle mixing, the 96-well plates were placed in an incubator at 25 °C (SANYA, Osaka, Japan). The plates were mixed well every 20 min. After 2 h, 100 μL of EPC cell suspension (10^6^ cells/well) was added to each well and cultured at 25 °C for 48 h. In accordance with the cytopathic effect (CPE) results, the serum neutralizing antibody titers were calculated using the Reed–Muench method [39]. All experiments were repeated three times.

### 2.13. LMBV Challenge

At 45 dpi, each group of largemouth bass (*n* = 20) was intraperitoneally injected with 100 μL of 3.47 × 10^6^ TCID_50_/mL LMBV. In addition, healthy largemouth bass (*n* = 20) was intraperitoneally injected with 100 μL sterile PBS as a control. The number of deaths in each group was recorded daily for 10 days, and the dead fish were removed. The RPS of the vaccine was calculated as follows: RPS = [1 − (inoculation group mortality%/control group mortality%)] × 100. The experiment was repeated three times.

### 2.14. Data Analysis

Data were analyzed using one-way analysis of variance (ANOVA) in the SPSS software (version 19.0; IBM Corp., Armonk, NY, USA). Significance was assessed using the Duncan multiple range test, and differences were considered significant at *p* < 0.05.

## 3. Results

### 3.1. MCP Codon Optimization

**Figure 1:**
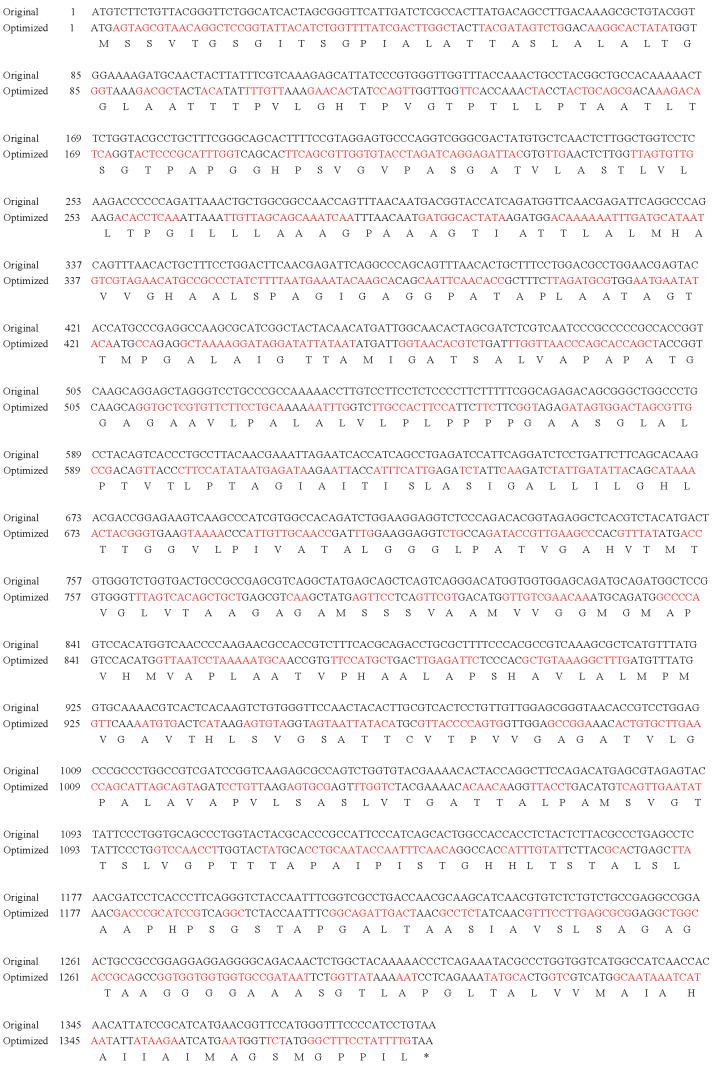
Codon optimization of the LMBV *MCP* nucleotide sequence. Original: the original sequence of LMBV-*MCP*; optimized: the optimized LMBV-*OMCP* sequence. The red tag was the optimized codon. The optimization rate was 69.76%. MCP, major coat protein; LMBV, largemouth bass virus.

The *MCP* gene sequence was optimized based on the *S. cerevisiae* expression system codon preferences while maintaining the amino acid sequence; 69.76% of *MCP* codons were optimized (marked in red in Figure 1). The optimized sequence was named *OMCP*. The CAI of *OMCP* was 0.79 (that of *MCP* was 0.65), and the GC content was 42.98% (that of *MCP* was 55.32%). The alignment of the sequences before and after optimization is shown in Figure 1.

### 3.2. The Recombinant Protein Was Displayed on the Surface of S. cerevisiae

**Figure 2:**
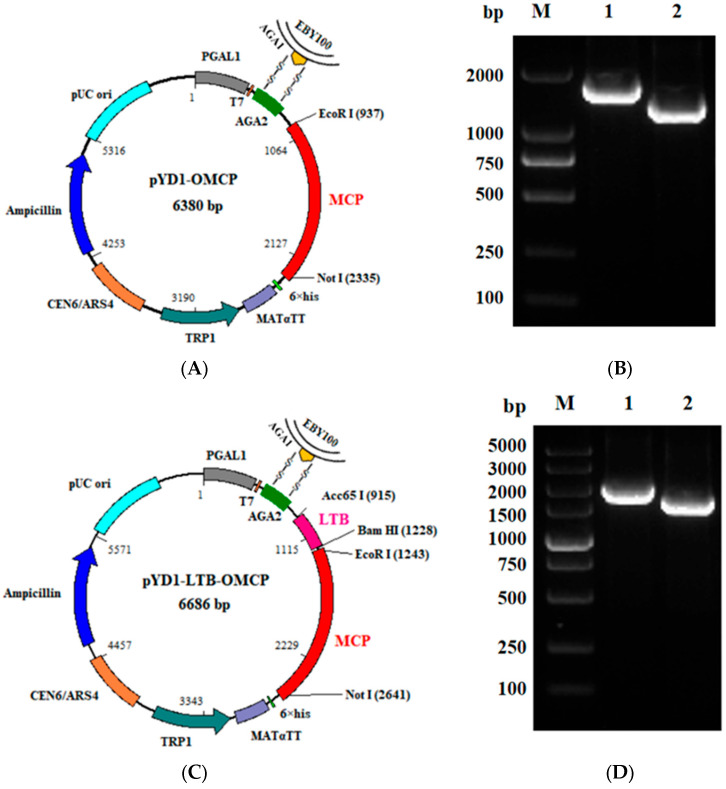

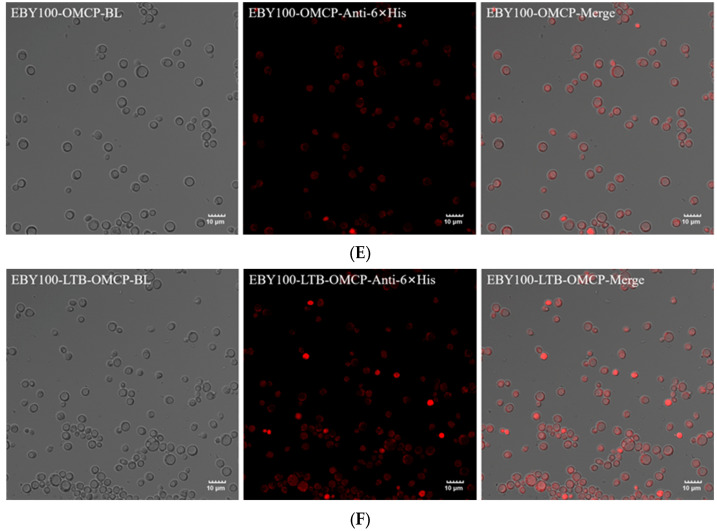
The yeast surface displayed pYD1-*OMCP* and pYD1-*LTB*-*OMCP*. (**A**) The pYD1-*OMCP* recombinant protein was displayed on the surface of EBY100 yeast cells through the disulfide bond between AGA2 and AGA1. (**B**) EBY100-OMCP positive yeast was identified using PCR. M: marker 2000 bp; 1: pYD1 universal primers; 2: *OMCP* specific primers. (**C**) The pYD1-*LTB*-*OMCP* recombinant protein was displayed on the surface of EBY100 yeast cells through the disulfide bond between AGA2 and AGA1. (**D**) EBY100-LTB-OMCP positive yeast was identified using PCR. M: marker 5000 bp; 1: pYD1 universal primers; 2: *LTB*-*OMCP*-specific primers. (**E**) The expression of the EBY100-OMCP recombinant protein was analyzed by immunofluorescence. Anti-mouse 6×His was used as the primary antibody, and goat anti-mouse IgG-H&L (Alexa Fluor 594) was used as the secondary antibody. EBY100-OMCP yeast appeared red under excitation light. Scale: 20 μm. (**F**) The expression of EBY100-LTB-OMCP recombinant protein was analyzed by immunofluorescence. EBY100-LTB-OMCP yeast appeared red under excitation light. Scale: 10 μm. LTB, heat-labile enterotoxin; AGA, a-agglutinin.

Based on the a-agglutinin yeast display system [40], the expression vector pYD1 was used to display recombinant proteins pYD1-*OMCP* and pYD1-*LTB*-*OMCP* on the surface of *S. cerevisiae*. The *OMCP* and *LTB*-*OMCP* genes were cloned into the C-terminus of aga2 in the pYD1 vector to obtain the expression vectors. After the vectors were transformed into yeast cells, the yeast aga1 protein was connected to the aga2-OMCP or aga2-LTB-OMCP through a disulfide bond (Figure 2A,C). Using the recombinant yeast EBY100-OMCP and EBY100-LTB-OMCP as templates, respectively, the target fragments pYD1-*OMCP* (universal primer: 1770 bp, specific primer: 1391 bp) and pYD1-*LTB*-*OMCP* (universal primer: 2076 bp, specific primer: 1716 bp) could be amplified (Figure 2B,D). With 6 × His as the detection antigen, the recombinant yeast EBY100-OMCP and EBY100-LTB-OMCP could be detected via red fluorescence (Figure 2E,F)

### 3.3. Recombinant Yeast Was Detected in the Second Intestine

**Figure 3:**
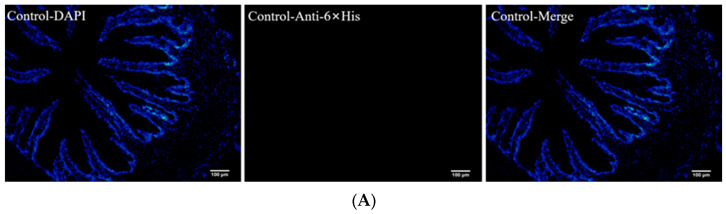

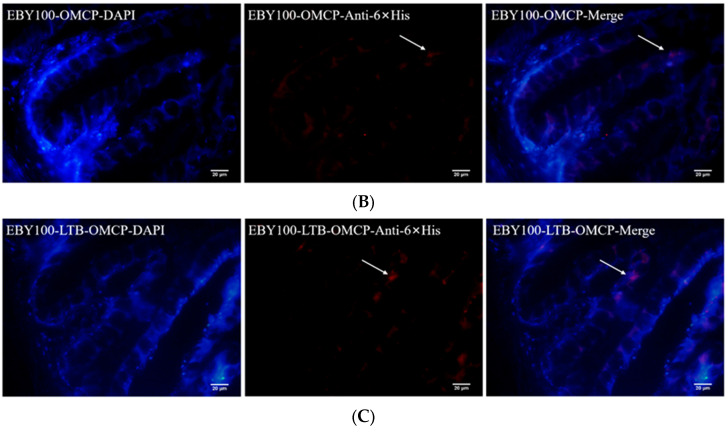
Detection of the antigens in the second intestine of largemouth bass. A total of 100 μL of PBS, EBY100-OMCP (1 × 10^7^ CFU/mL), and EBY100-LTB-OMCP (1 × 10^7^ CFU/mL) were intragastrically administered to largemouth bass separately. *n* = 3. After 24 h, the intestinal tissues were taken to prepare frozen sections. Anti-6×His was used as the primary antibody, Alexa Fluor 594 was used as the secondary antibody, and the nucleus was stained using DAPI. (**A**) PBS treated, no red fluorescence signal. Scale: 100 μm. (**B**) EBY100-OMCP treated with a red fluorescence signal. Scale: 20 μm. (**C**) EBY100-LTB-OMCP treated with a red fluorescence signal. Arrows: the detected antigen. scale: 20 μm. PBS, phosphate-buffered saline; CFU, colony-forming units; DAPI, 4′,6-diamidino-2-phenylindole.

The vaccines and PBS were administered via oral gavage to largemouth bass. The recombinant yeast was detected in the midgut using immunohistochemical staining. Red fluorescence was detected in the intestines of the vaccine-treated fish (Figure 3B,C) but not in the PBS-treated fish (Figure 3A).

### 3.4. Safety Evaluation of Oral Vaccine

**Figure 4:**
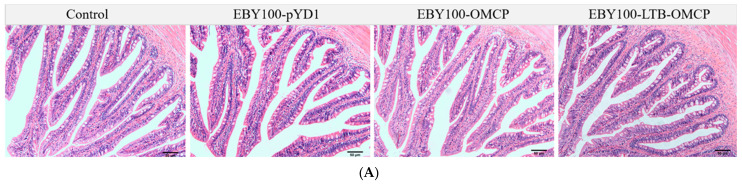

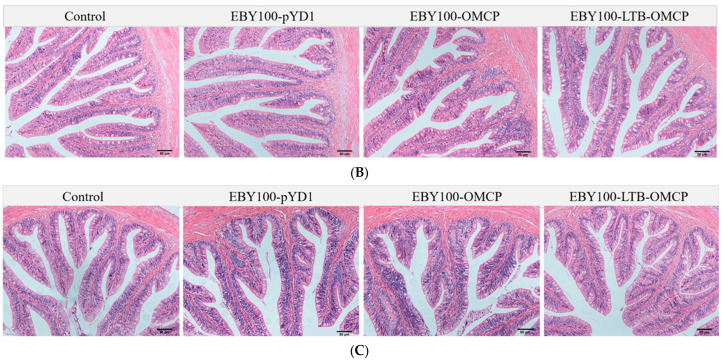
Microscopic observation of intestinal tissues of largemouth bass. The foregut, midgut, and hindgut samples of each group were fixed in 4% paraformaldehyde universal tissue fixative. After hematoxylin and eosin (H&E) staining, the intestinal tissue morphology was observed under an optical microscope. *n* = 3. (**A**) Foregut; (**B**) midgut; (**C**) hindgut. Scale: 50 μm.

The safety of the yeast vaccines was evaluated by the feeding behavior, death rate, and intestinal microscopic observation of largemouth bass during immunization. During the immunization period, the feeding and activity behaviors of each group were normal, and no signs of disease or death occurred. The intestinal tissues of the largemouth bass were observed under an optical microscope. As shown in Figure 4, no pathological changes were observed.

### 3.5. Detection of Immune-Related Enzyme Activity

**Figure 5:**
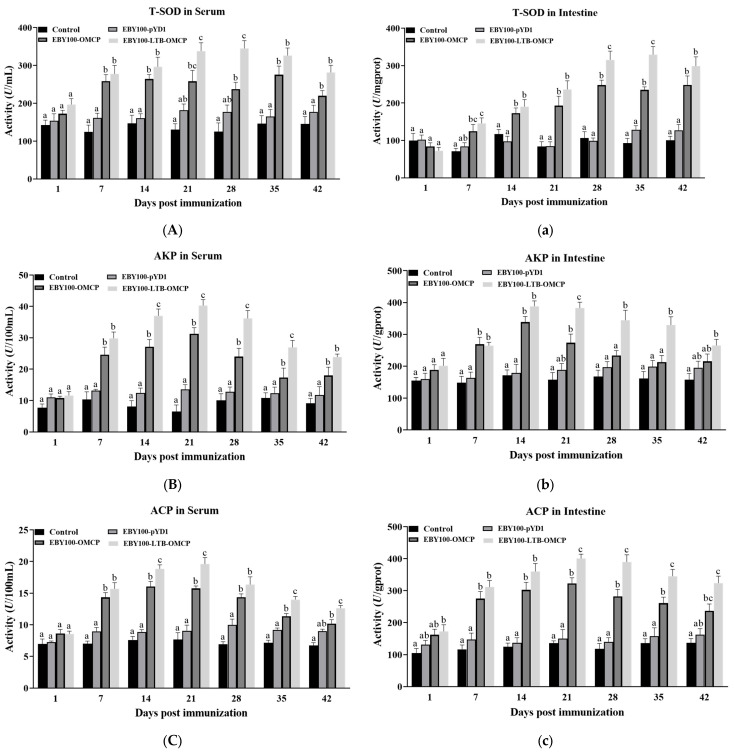

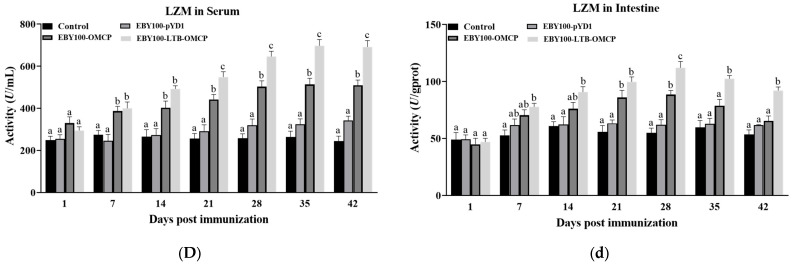
Effects of oral vaccines on the activities of innate immune enzymes of largemouth bass (means ± SEM, *n* = 5). The enzyme activities were detected using their corresponding kits. (**A**–**D**) The activities of T-SOD, AKP, ACP, and LZM in serum. (**a**–**d**) The activities of T-SOD, AKP, ACP, and LZM in midgut mucus. Different superscript letters in each group (a–c) indicate significant variations according to the Kruskal–Wallis statistics at 95% significance, followed by Dunn’s test with Bonferroni adjustment as the post hoc test (*p* < 0.05). T-SOD, superoxide dismutase; AKP, alkaline phosphatase; ACP, acid phosphatase, LZM, lysozyme.

Enzyme activities were determined using commercial kits. The T-SOD activity in the serum and intestinal mucus are shown in Figure 5A,a. Compared with the control group, the activity of T-SOD in the immunized groups was significantly higher (*p* < 0.05 or *p* < 0.01). In the serum, the T-SOD activity of the EBY100-OMCP group was significantly increased at 7 dpi (258.5 ± 24.4 U/mL) and then remained constant. The T-SOD activity in the EBY100-LTB-OMCP group peaked at 28 dpi (345.1 ± 20.5 U/mL) and then decreased. The AKP activities in the serum and intestinal mucus are shown in Figure 5B,b. The AKP activity in the serum of the immunized group increased significantly at 7 dpi and peaked at 21 dpi (EBY100-OMCP: 31.3 ± 1.95 U/100 mL; BY100-LTB-OMCP: 40.3 ± 1.86 U/100 mL). The AKP activity in the intestinal mucus reached a peak at 14 dpi (EBY100-OMCP: 338.6 ± 17.44 U/gprot; BY100-LTB-OMCP: 388.3 ± 17.00 U/gprot) and then gradually decreased. The ACP activity in the serum and intestinal mucus began to decrease after 21 dpi in both vaccine groups (Figure 5C,c). The LZM activities are shown in Figure 5D,d. In the serum, the LZM activity of the two vaccine groups increased steadily, with the highest values of 512.8 ± 30 U/gprot (EBY100-OMCP group) and 696.6 ± 17.00 U/gprot (EBY100-LTB-OMCP group). The LZM activity in the intestinal mucus of the two vaccine groups began to decrease after 28 dpi. Throughout the immunization period, the immune enzyme activity of the EBY100-LTB-OMCP group was superior to that of the EBY100-OMCP group, especially at 21 dpi, 28 dpi, and 35 dpi (*p* < 0.05 or *p* < 0.01).

### 3.6. Expression of Immune-Related Genes

**Figure 6:**
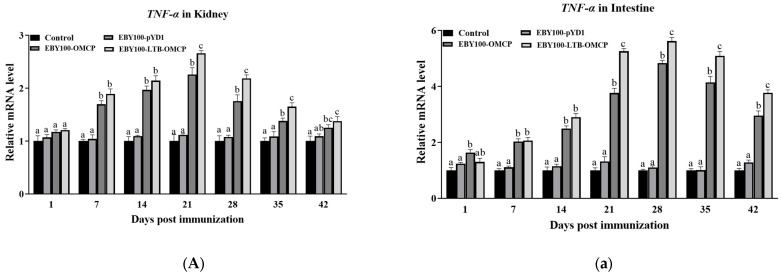

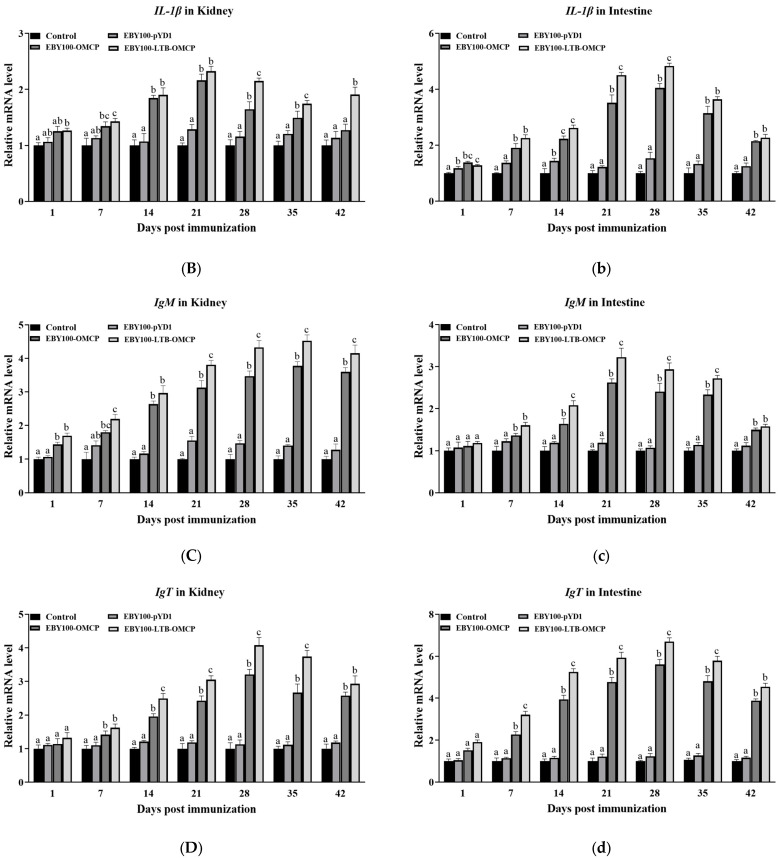
Effects of oral vaccines on immune gene expression in largemouth bass (means ± SEM, *n* = 5). The relative qRT-PCR gene expression analysis was performed using the 2^−ΔΔCT^ method. (**A**–**D**) The expression levels of *IL-1β*, *TNF-α*, *IgM*, and *IgT* genes in the head kidney. (**a**–**d**) The expression of *IL-1β*, *TNF-α*, *IgM* and *IgT* gene in intestine. Different superscript letters in each group (a–c) denote significant variations according to the Kruskal–Wallis statistics at 95% significance, followed by Dunn’s test with Bonferroni adjustment as the post hoc test (*p* < 0.05). TNF-α, tumor necrosis factor alpha; IL-1β, interleukin 1 beta; IgM, immunoglobulin M; IgT, immunoglobulin T; qRT-PCR, quantitative real-time reverse transcription PCR.

The expression levels of immune genes in the head kidney and intestine were analyzed using qRT-PCR, as shown in Figure 6. Compared with the control group, the expression levels of the four genes (*TNF-α*, *IL-1β*, *IgM*, and *IgT*) in the immunized groups showed a trend of increasing first and then gradually returning to the basic level. The expression of *TNF-α* and *IL-1β* in the head kidney was the highest at 21 dpi (EBY100-OMCP group: 2.3-fold, 2.2-fold; BY100-LTB-OMCP group: 2.7-fold, 2.3-fold) (Figure 6A,B), and the highest expression in the intestine was at 28 dpi (EBY100-OMCP group: 4.8-fold, 4.0-fold; BY100-LTB-OMCP group: 5.6-fold, 4.8-fold) (Figure 6a,b). The expression of *IgM* in the head and kidney (EBY100-OMCP group: 3.8-fold; EBY100-LTB-OMCP group: 4.3-fold) was higher than that in the intestine (EBY100-OMCP group: 2.6-fold; BY100-LTB-OMCP group: 3.2-fold), and the head kidney maintained a high level of *IgM* for a long time (28–42 dpi) (Figure 6C,c). *IgT* was more highly expressed in the intestine (EBY100-OMCP group: 5.6-fold; BY100-LTB-OMCP group: 6.7-fold) than in the head kidney (EBY100-OMCP group: 3.2-fold; BY100-LTB-OMCP group: 4.1-fold) (Figure 6D,d).

### 3.7. Serum Antibody Levels

**Figure 7:**
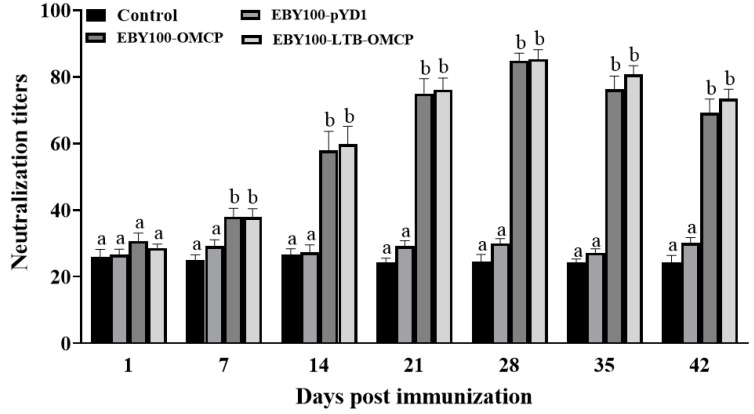
Determination of serum neutralizing antibody titer of largemouth bass. A total of 50 μL of serum was mixed with 50 μL 10^3 ^TCID_50_/mL LMBV virus and inoculated into EPC cells to observe the cytopathic effect (means ± SEM, *n* = 5). Different superscript letters in each group (a–b) denote significant variations according to the Kruskal–Wallis statistics at 95% significance, followed by Dunn’s test with Bonferroni adjustment as the post hoc test (*p* < 0.05).

The specific immune response induced by the vaccines was evaluated using the serum neutralization titer. The serum was mixed with the LMBV virus, and the mixture was inoculated into EPC cells to observe the CPE. The serum antibody levels are shown in Figure 7. For the immunized fish, the antibody titer peaked at 28 dpi. The antibody titer of both vaccine groups was 1:85, which was significantly higher than that of the control group and the pYD1 group (*p* < 0.05).

### 3.8. Oral Vaccine Protection against LMBV

**Figure 8:**
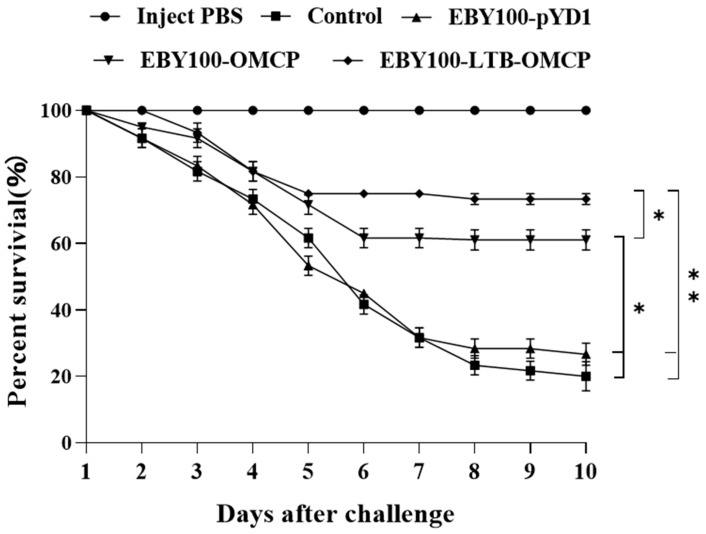
The survival rate of largemouth bass against LMBV infection. Each group was intraperitoneally injected with 100 μL of PBS or LMBV (3.47 × 10^6^ TCID_50_/mL). *n* = 20, with death recorded daily. After LMBV infection, the cumulative death in each group was calculated using the log-rank (Mantel–Cox) test (* *p* < 0.05, ** *p* < 0.01).

Largemouth bass were infected with LMBV (3.4 × 10^6^ TCID_50_/mL). The results of the vaccine protection experiment are shown in Figure 8. The control group and EBY100-pYD1 group injected with LMBV began to die at day 2 post-infection, and death peaked on days 5, 6 and 7. The control group’s final survival rate was 20 ± 4.41%, while the EBY100-pYD1 group’s final survival rate was 26.67 ± 3.33%. The EBY100-OMCP group began to die at 2 days post-infection and remained stable after the 6th day. On day 10 post-infection, the survival rate was 61.67 ± 2.89%. The EBY100-LTB-OMCP group mainly died 3–5 days after infection, and the final survival rate was 73.33 ± 1.67%. The RPS of the EBY100-LTB-OMCP group was as high as 66.66%, and the EBY100-OMCP group had a 52.10% protective effect. The control fish injected with PBS did not die during this period.

## 4. Discussion

Yeast surface display technology is a rapidly developing eukaryotic protein expression system [41]. As a general technology platform, it can be used to develop vaccines for pathogens such as bacteria, viruses, fungi, and parasites. When the hemolysin protein *HL*1 of *Vibrio harveyi* was displayed on the surface of *S. cerevisiae*, the recombinant yeast had a significant protective effect on turbot (*Scophthalmus maximus*) and flounder (*Pleuronectiformes*) [42]. Displaying the full-length receptor binding domain (RBD) of the SARS-CoV2 spike protein on the surface of *S. cerevisiae* EBY100 was found to be capable of inducing significant humoral and mucosal immunity in mice at the laboratory level [43]. In this study, vaccines were prepared by displaying the MCP protein of LMBV on the surface of *S. cerevisiae*. The MCP protein was directly exposed on the surface of yeast cells, which increased the opportunity for antigen recognition.

The optimized (*OMCP*) CAI index and GC content are more suitable for yeast expression systems [38]. To improve the expression of MCP protein in yeast, the codons of *MCP* were optimized in this study. In addition, mucosal immune adjuvants can enhance the protective effect of vaccines. After the fusion of LTB and GFP, the ability of the carp (*Cyprinidae*) hindgut to take up foreign proteins was higher than that of GFP alone, and LTB-GFP can exist in the large macrophages of the intestinal mucosa [44]. The LTB-NS1Δ63 vaccine against the Japanese encephalitis virus (JEV) protected 90% of mice from death, much higher than that of the NS1Δ63 vaccine (55% of survival rate) [45]. In the present study, pYD1-*LTB*-*OMCP* was successfully constructed by fusing *LTB* and *OMCP*. To the best of our knowledge, this is the first report on the combination of *S. cerevisiae* display technology and mucosal immune adjuvant LTB.

The second segment of the fish intestine is considered to be the main site of antigen uptake and plays a major immune role [46,47]. To test whether the antigen can be delivered to the site of action, the marker antigen GFP was expressed in *P. pastoris*. After intragastric administration to stomachless teleost fish (*Cyprinidae*) and stomach teleost fish (*Oncorhynchus mykiss*), it was observed that the GFP signal in the second intestine of teleost fish with a stomach was lower than that of stomachless teleost fish [48]. Subsequently, a study showed that the ORF131 protein of cyprinid herpesvirus 3 (CyHV3) expressed by *S. cerevisiae* EBY100 could be detected in the second intestine of carp [49]. In this study, the red fluorescence of the recombinant yeasts could be detected in the second intestine of the immunized group. This indicated that *S. cerevisiae* EBY100 could deliver complete antigens to the active site of the intestines, which is an important prerequisite for an effective oral vaccine.

T-SOD, AKP, ACP, and LZM are generally selected as indicators to evaluate the immune status and disease resistance of fish. Previously, it was reported that the oral double-targeted DNA vaccine of *Vibrio mimicus* could significantly increase the activity of SOD and LZM in the serum and intestinal mucus of grass carp (*Ctenopharyngodon idella*) [50]. The oral *Bacillus subtilis* vaccine expressing VP56 of grass carp reovirus II (GCRVII) markedly increased the activity of immune-related enzymes in the serum and intestine of immunized grass carp [35]. Here, we examined the levels of T-SOD, AKP, ACP, and LZM in the serum and intestinal mucus of largemouth bass. The vaccine group maintained a high level of enzyme activity during immunization (7–35 dpi). AKP and ACP are involved in the process of cell digestion of antigens [51]. T-SOD can eliminate superoxide radicals produced by phagocytosis [52]. LZM can also activate leukocytes and phagocytic cells [53]. The general increase in these four enzyme activities suggested enhanced phagocytosis in the body. The vaccine containing the MCP antigen was swallowed, thereby improving the innate immune level of the fish. In the study of an LMBV immersion vaccine, the increase in AKP activity was not obvious [31]. However, in this study, the AKP activity of the vaccine group increased significantly, and the AKP activity of the empty yeast control group (EBY100-pYD1) also increased slightly (*p* > 0.05). Thus, the glucan and other components in the yeast cell wall might play a role [54].

To further evaluate the immune effect of the vaccines, we focused on the genes of innate immunity (*TNF-α*, *IL-1β*) and adaptive immunity (*IgM*, *IgT*). *TNF-α* and *IL-1β* are important cellular inflammatory factors [55]. In this study, the mRNA levels of *IL-1β* and *TNF-α* in the head kidney and intestine were significantly upregulated in the vaccine groups. *TNF-α* was significantly upregulated earlier than *IL-1β*. It was possible that *TNF-α*, one of the first cytokines produced in response to viral infection, could trigger pro-inflammatory cascades, including *IL-1β* [34,56]. The expression of *TNF-α* and *IL-1β* in a previous LMBV DNA vaccine report peaked at 7 dpi, earlier than the 21 dpi observed in this study [18]. This might be because injection inoculation could cause a faster immune response in the body. Among the three immunoglobulins present in fish, *IgM* plays a major role in systemic immunity [57], and *IgT*/*Z* plays a key role in mucosal immunity [32,58,59]. In this study, *IgM* was significantly upregulated in the head kidney and intestine of largemouth bass compared to controls. *IgM* expression was continuously high (28–42 dpi) in the head kidney of the vaccinated groups. This was similar to the *IgM* expression in gibel carp (*Carassius auratus gibelio*) induced by the cyprinid herpesvirus 2 (CyHV2) yeast oral vaccine [60]. As for *IgT*, our results showed that the gene was highly expressed in the head kidney and intestine of the immunized groups, as expected, and the expression level was higher in the intestine. These results showed that oral vaccines could effectively trigger intestinal mucosal immunity, represented by *IgT* upregulation [49]. The expression level of *IgT* in the intestines of rainbow trout (*Oncorhynchus mykiss*) was stronger than that in the head kidney after inoculation with the EBY100 yeast vaccine against infectious hematopoietic necrosis virus (IHNV) [61], which was consistent with the results of this study. Compared with the EBY100-OMCP group, the upregulation of immune genes in the EBY100-LTB-OMCP group was more obvious, indicating that the presence of LTB was important for producing a strong immune response [26].

Neutralizing antibodies play a central role in clearing pathogens [62]. In this study, vaccination induced high titers of anti-LMBV antibodies (1:85) in largemouth bass serum. Interestingly, compared with that produced by the ordinary vaccine EBY100-OMCP, the antibody level induced by the EBY100-LTB-OMCP vaccine was not significantly higher (*p* > 0.05), which differed from the results of previous studies [45,63]. However, serum antibody levels are not necessarily associated with vaccine protection [64]. In response to LMBV infection, the RPS of the EBY100-LTB-OMCP vaccine reached 66.66%, which was significantly higher than that of the EBY100-OMCP vaccine (52.10%) (*p* = 0.0379). Therefore, mucosal immunity stimulated by LTB might play a significant role in the anti-LMBV effect.

## 5. Conclusions

In conclusion, this study described highly safe LMBV oral vaccines based on yeast surface display technology. The vaccines could not only induce systemic immunity in largemouth bass but also effectively promote the activation of intestinal mucosal immunity. When challenged by LMBV infection, the mortality rates in the vaccine groups were significantly reduced. The vaccine preparation method has broad prospects for preparing oral vaccines for other pathogens. However, more samples are needed to verify the safety of the vaccine. In order to prepare more effective vaccines, the parameters such as the immune dose of the vaccine, the vaccination regimen, and the selection of immune adjuvants need to be further explored. Cytokines (IL-1β, IL-8, G-SCF, IFN-γ, etc.) have been shown to have adjuvant effects. The combination of multiple cytokine adjuvants may improve the immune effect of a single adjuvant in this study.

## Figures and Tables

**Table 1:** Primers used in the study.

Name	Sequence (5′-3′)	Size (bp)	Reference	Usage
pYD1	F-AGTAACGTTTGTCAGTAATTGC	399	Designed	PCR
	R-GTCGATTTTGTTACATCTACAC			
*OMCP*	F-GGATCCAGTGTGGTGGAATTCATGAGTAGCGTAACAGGC	1389	Designed	PCR
	R-GCCCTCTAGACTCGAGCGGCCGCCACAAAATAGGAAAGCCC			
*ltb*	F-GGATCCAGTGTGGTGGGTACCCCTCAGTCTATTACAGAGC	306	Designed	PCR
	R-TGAATTCCACCACACTGGATCCGTTTTCCATACTGATTGCCG			
*TNF-α*	F-ACTTCGTCTACAGCCAGGCA	105	[18]	qRT-PCR
	R-AGTAACGCGAGACCCTGTGG			
*IL-1β*	F-TGGTGGAAAACAGCATGGAGC	95	[18]	qRT-PCR
	R-AGGGTGCACGTAGTTCGACA			
*IgM*	F-GACTGGAGTGGCGGAAAGTGGAGG	133	[31]	qRT-PCR
	R-TTTCATCTTCTACAAACGCAGACAACGG			
*IgT*	F-GAAGGTCAACAACGCTGAGTG	248	[32]	qRT-PCR
	R-TGTTGCTGGTCACATCTAGTCC			
*β-actin*	F-CAGGATGCAGAAGGAGATCACA	151	[18]	qRT-PCR
	R-CTCCTGCTTGCTGATCCACAT			

Notes: Red front: restriction sites; underline: homologous arm sequence. OMCP, optimized major capsid protein; LTB, heat-labile enterotoxin; TNF-α, tumor necrosis factor alpha; IL-1β, interleukin 1 beta; IgM, immunoglobulin M; IgT, immunoglobulin T; qRT-PCR, quantitative real-time reverse transcription PCR.

## Data Availability

The datasets generated for this study are available upon request to the corresponding author.

## Supplementary Materials

The following supporting information can be downloaded at: https://www.mdpi.com/article/10.3390/ani13071183/s1, Figure S1: Vaccination/challenge and sampling schedule.
