# Supplementary figures and images for: Oral Vaccination of Largemouth Bass (Micropterus salmoides) against Largemouth Bass Ranavirus (LMBV) Using Yeast Surface Display Technology
Source: Animals (Basel). 2023 Mar 28;13(7):1183. doi: 10.3390/ani13071183 (PMC10093309; doi:10.3390/ani13071183)

Figure S1

Vaccination/challenge and sampling schedule.

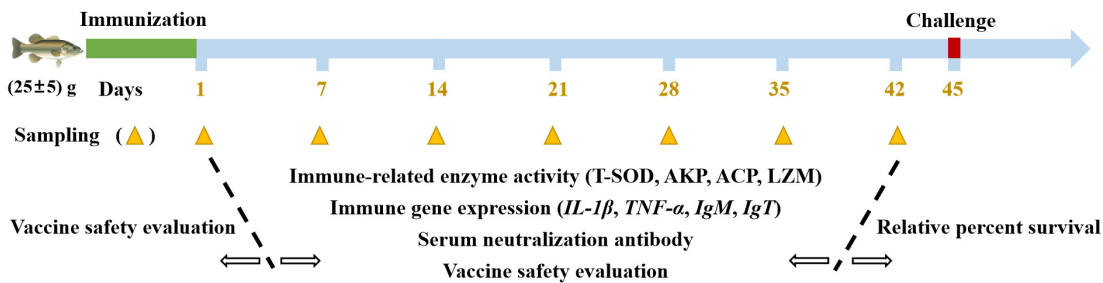

Supplement: Supplementary file 1 [file animals-13-01183-s001.zip › animals-2257901-supplementary.pdf]
